# Evaluation of Bacteriophage Cocktail on Septicemia Caused by Colistin-Resistant *Klebsiella pneumoniae* in Mice Model

**DOI:** 10.3389/fphar.2022.778676

**Published:** 2022-02-07

**Authors:** Aprajita Singh, Alakh Narayan Singh, Nisha Rathor, Rama Chaudhry, Sudhir Kumar Singh, Gopal Nath

**Affiliations:** ^1^ Department of Microbiology, Institute of Medical Sciences, Banaras Hindu University, Varanasi, India; ^2^ Department of Microbiology, All India Institute of Medical Sciences, New Delhi, India

**Keywords:** *Klebsiella pneumoniae*, septicemia, phage cocktail, endotoxin, IL-6

## Abstract

**Objective:** The emergence of resistance against last-resort antibiotics, carbapenem and colistin, in *Klebsiella pneumoniae* has been reported across the globe. Bacteriophage therapy seems to be one of the most promising alternatives. This study aimed to optimize the quantity and frequency of bacteriophage cocktail dosage/s required to eradicate the *Klebsiella pneumoniae* bacteria in immunocompetent septicemic mice.

**Methods:** The three most active phages ɸKpBHU4, ɸKpBHU7, and ɸKpBHU14 characterized by molecular and TEM analyses were in the form of cocktail and was given intraperitoneally to mice after inducing the septicemia mice model with a constant dose of 8 × 10^7^ colony-forming unit/mouse (CFU/mouse) *Klebsiella pneumoniae*. After that, the efficacy of the phage cocktail was analyzed at different dosages, that is, in increasing, variable, constant, and repeated dosages. Furthermore, interleukin-6 and endotoxin levels were estimated with variable doses of phage cocktail.

**Results:** We have elucidated that phage therapy is effective against the *Klebsiella pneumoniae* septicemia mice model and is a promising alternative to antibiotic treatments. Our work delineates that a single dose of phage cocktail with 1 × 10^5^ plaque-forming unit/mouse (PFU/mouse) protects the mice from fatal outcomes at any stage of septicemia. However, a higher phage dosage of 1 × 10^12^ PFU/mice is fatal when given at the early hours of septicemia, while this high dose is not fatal at the later stages of septicemia. Moreover, multiple repeated dosages are required to eradicate the bacteria from peripheral blood. In addition, the IL-6 levels in the 1 × 10^5^ PFU/mouse group remain lower, but in the 1 × 10^12^ PFU/mouse group remains high at all points, which were associated with fatal outcomes.

**Conclusion:** Our study showed that the optimized relatively lower and multiple dosages of phage cocktails with the strict monitoring of vitals in clinical settings might cure septicemia caused by MDR bacteria with different severity of infection.

## Introduction

Sepsis is defined as a syndromic response to infection. It may be a final common pathway to death from many infectious diseases worldwide. As per an estimate, 20% of all global deaths are attributed to septicemia (Rudd et al., 2020). Approximately, 85% of sepsis cases and related deaths occur in developing countries. Schooley et al. from the United States have published the much-admired human case report. They have successfully used a personalized bacteriophages cocktail on a 68-year-old terminally ill, diabetic patient with necrotizing pancreatitis complicated with MDR *A. baumannii* infection (Schooley et al., 2017). However, specific unresolved issues such as optimization of safe dosages (quantity and frequency), modes of administration, pharmacokinetics, pharmacodynamics, the multiplicity of infection and valency, the characterization of phages and deployment, and the emergence of bacteriophage resistance during therapy must be worked out thoroughly before clinical trials. We have to discern the quantity and number of the cocktail doses with different stages of septicemia in various age-groups of the patients. Reports indicate that the administration of a cocktail of antibiotic disrupting cell walls in severe septicemia often results in a fatal outcome due to sudden massive lysis of the bacteria and release of a massive amount of endotoxin (Prins et al., 1994; Skorup et al., 2020). The bacteriophages also kill bacteria by disrupting the cell wall, so their use may have similar consequences if the dosage is not optimized. Even Schooley et al*.* used the empirical doses and frequency of bacteriophage cocktail with strict monitoring of the patient’s vitals (Schooley et al., 2017).

Therefore, the safe dosage for the different stages of septicemia must be decided in the preclinical model. *Klebsiella pneumoniae* is a member of the *Enterococcus faecium*, *Staphylococcus aureus*, *Klebsiella pneumoniae*, *Acinetobacter baumannii*, *Pseudomonas aeruginosa*, and *Enterobacter* species (ESKAPE) group of bacteria, notoriously known for their multi-/pan-drug resistance status. The “last-resort” antimicrobial agent to fight MDR *K. pneumoniae* infections is colistin, which causes nephrotoxicity (Arnold et al., 2011). However, the recent reports of colistin-resistant *K. pneumoniae* further limit the antimicrobial options, resulting in high mortality associated with the infection (Capone et al., 2013). Therefore, bacteriophage therapy is emerging as one of the promising alternative approaches for treating even colistin- and carbapenem-resistant *K. pneumoniae* (C-C-RKp) infections. Therefore, we carried out this study to optimize the quantity and frequency of the bacteriophage cocktail dosage to treat the various severity/duration of septicemia caused by *K. pneumoniae* in a mouse model.

## Materials and Methods

### Experimental Animal and Bacterial Strain

The *K. pneumoniae* strain used in the present study was isolated from the endotracheal tube of a patient admitted to the intensive care unit (ICU) of the Sir Sunderlal Hospital (university hospital) of Banaras Hindu University Varanasi, India. The antibiotic sensitivity test was performed in our laboratory as a routine diagnostic procedure in the Department of Microbiology, Institute of Medical Sciences, Banaras Hindu University. Therefore, consent from the patient could not be obtained. The carbapenem-/colistin-resistant strain was further assigned a laboratory code KpnBHU101 and used for the bacteriophage screening and generation of the septicemia mice model. All the animal experiments were performed on 6- to 8-week-old immunocompetent inbred Swiss albino mice, weighing 20–25 g. The mice were reared in the Institute of Medical Sciences Central Animal House, Banaras Hindu University, Varanasi. The Institutional Ethics Committee for animals permitted the protocol for the proposed study, which was carried out from December 2018 to February 2020 (Reference no. Dean/2015/CAEC/99T).

### Antibiotic Sensitivity Testing

The minimum inhibitory concentration of imipenem, meropenem, levofloxacin, tigecycline, polymyxin-B, and polymyxin-E (colistin) against *K. pneumoniae* KpnBHU101 was carried out by using the broth dilution method following the recommendation of the Clinical and Laboratory Standards Institute (Patel et al., 2015; Humphries et al., 2018). The AMR profile of the KpnBHU101 was mentioned in the Supplementary Table S1. All the antibiotic discs and powder were procured from Hi-Media Pvt. Ltd., Mumbai, India. A known reference strain of *K. pneumoniae* (ATCC 13883) was used as the positive control.

### Isolation and Purification of Phages

Phages were isolated from different water sources, namely, Sir Sundar Lal hospital sewer (BHU), Durgakund pond, and the Ganga River, Varanasi. The collected water samples were centrifuged at 10,000 × *g* for 10 min, and the supernatant was filtered through a 0.22-µm filter. Phage propagation followed the method as described elsewhere (Taha et al., 2018; Manohar et al., 2019)*.* One milliliter of filtered water sample was incubated with KpnBHU101 at 37°C for 3–4 h and added to 50 ml Luria Bertani (LB) broth and incubated at 37°C overnight. The suspension was centrifuged thrice at 10,000 × *g* for 10 min. The bacteriophage titer in the supernatant was enumerated, as described previously (Adams, 1959).

The harvested fluid was subjected to membrane dialysis against 30% polyethylene glycol (PEG 6000) in 2.5 M NaCl for 18–20 h, followed by washing thrice with phosphate-buffered saline (PBS) at 4°C for purification and concentration of phages, as described elsewhere (Gangwar et al., 2021). The endotoxin estimation of purified phages was done using an ELISA kit (Thermo Scientific™ Pierce™ LAL Chromogenic Endotoxin Quantitation Kit). Finally, the purified phage particles were preserved at −20°C for further use.

### Antibacterial Activity of Purified Phages

Fifty different phage isolates were screened for their antibacterial activity on 70 clinical isolates of *K. pneumoniae*, and among 70 clinical isolates, 53 were carbapenem-resistant. The lawn culture of bacteria with an approximate concentration of 6 × 10^8^ CFU/ml (∼2.0 McFarland standard) was made on Mueller Hinton Agar (MHA). Ten microliters of each phage with a concentration 10^9^ PFU/ml was spotted on the MHA plate. The MHA plates were observed for the clear zone after 16–18 h incubation at 37°C. The three most active phages, ɸKpBHU4, ɸKpBHU7, and ɸKpBHU14, were selected for further characterization.

### Characterization of Bacteriophage ɸKpBHU4, ɸKpBHU7, and ɸKpBHU14

#### Transmission Electron Microscope Analysis

Phage particles ɸKpBHU4, ɸKpBHU7, and ɸKpBHU14 with a concentration 1 × 10^10^ PFU/ml were filtered through 0.22-μm PVDF syringe filter. Furthermore, the phage particles were centrifuged at 25,000 ×*g* for 75 min, and the pellet was washed thrice in 0.1 M ammonium acetate, pH 7.0. Finally, the supernatant was decanted and resuspended pellet in ammonium acetate. Furthermore, 2% uranyl acetate was used for negative staining of samples on carbon-coated Formvar films and examined by transmission electron microscopy (model CRYO-TEM) (TALOS S, Thermo Scientific AIIMS, New Delhi, India).

#### Temperature and pH Sensitivity

Temperature and pH tolerance was determined as described earlier (Sadekuzzaman et al., 2017). In brief, the bacteriophages in equal volume were incubated in a water bath for 180 min at different temperatures 4, 20, 37, 50, 60, 70, and 80°C. Furthermore, enumeration of phages was done immediately after incubation. pH tolerance assay was performed as described previously (Zurabov and Zhilenkov, 2021).

#### Phage Killing Curve (Burst Size)

A one-step growth curve experiment was performed to estimate the burst size of the three most active phages. In brief, KpnBHU101 was grown in LB medium at 37°C until the optical density (OD) reached 0.6. Then, 1 mL of the culture was harvested by centrifugation at 4,000 × g for 5 min. Furthermore, the pellet was mixed with 0.1 ml of phage particle with multiplicity of infection (MOI) of 0.001. The phage–bacteria complex was collected after 10 min, resuspended in LB broth, and kept at 37°C. During the subsequent incubation, aliquots of 0.5 ml were taken at 5-min intervals for 120 min. One-step growth curve of each released phage from KpnBHU101 was plotted against time using GraphPad Prism 5.0.

#### Molecular Analysis of Phage DNA

The DNA of three phages ɸKpBHU4, ɸKpBHU7, and ɸKpBHU14 was extracted and purified from phage lysates using a QIAGEN^®^ Lambda Midi Kit (QIAGEN Inc., Valencia United States) according to the manufacturers’ protocol (Kesik-Szeloch et al., 2013).

##### Analysis by Restriction Digestion

The three selected phage DNAs were subjected to restriction digestion using enzyme EcoRI as per the manufacturers’ protocol (Thermo Scientific). The digested product was electrophoresed with 1% agarose gel at 80 V for 2 h at room temperature, and further imaging was done using a gel documentation system (BioRad, Universal Hood II, United States).

##### Analysis by Random Amplified Polymorphic DNA

The DNA templates of all the three phages used in the experiment were subjected to genotyping by RAPD-PCR (Czajkowski et al., 2015). The primer sequences used for RAPD PCR were 5′AGTTCAGAGTGC3′. The PCR amplification reactions were carried out in a thermal cycler (T100 Thermal Cycler, BIO-RAD, CA, United States).

### Phage Cocktail Preparation

The three different phages ɸKpBHU4, ɸKpBHU7, and ɸKpBHU14 with a broad spectrum lytic activity against 70 clinical *K. pneumoniae* were used for the cocktail preparation. A phage cocktail containing equal concentration and volume of the aforementioned three phages was prepared for the desired concentration.

### Lethal Dose_100_

A group of 5 mice was given an antibiotic-free diet. A volume of 100 μL of *K. pneumoniae* at a concentration of 8 × 10^7^ CFU/mouse through the intraperitoneal (IP) route was lethal for all the mice between 24 and 48 h (see Supplementary Table S2).

### Safety of Phage Cocktail

Intraperitoneal injection of 100 μL phage cocktail (1 × 10^12^ PFU/mouse, empirically) was given to a group of five mice. These mice were observed for a month for any disease development.

### Assessment of Microbiological and Clinical Efficacy of the Phage Cocktail

The phage cocktail was used for prophylactic and therapeutic purposes. The mice experiments were set up as per the following groupings, and each group contained five mice. The sickness of mice was graded based on the following features: 1: normal: no detectable abnormality; 2: slight illness: lethargy, ruffled fur; 3: moderate illness: severe lethargy, ruffled fur, and hunched back; 4: severe illness: aforementioned signs with exudative accumulation around eyes; and 5: death. The plan of the phage cocktail administration is shown in Table 1.

**TABLE 1:** Plan of the bacterial challenge and administration of phage cocktail at different time point.

S. No	Phage cocktail (PFU/mouse)	Phage cocktail administration	Phage cocktail and KpnBHU101 (simultaneous)	Phage cocktail (6 h before bacterial challenge)	Phage cocktail (6 h after bacterial challenge)	Phage cocktail (12 h after bacterial challenge)	Phage cocktail (24 h after bacterial challenge)
1	1 × 10^2^	–	–	–	✔	–	–
2	1 × 10^3^	–	✔	–	✔	–	–
3	1 × 10^4^	–	–	–	✔	–	–
4	1 × 10^5^	✔	✔	✔	✔	✔	✔
5	1 × 10^12^	–	–	–	✔	–	✔

The symbol (✔) represents phage and bacterial challenge given.

#### Administration of Phage Cocktail in Increasing Dosage

Three groups, comprising five mice in each, were challenged with 100 μL of KpnBHU101 (8 × 10^7^ CFU/mouse) through the intraperitoneal route. After 6 h of bacterial challenge, 100 μL of phage cocktail containing 1 × 10^2^ PFU/mouse, 1 × 10^3^ PFU/mouse, and 1 × 10^4^ PFU/mouse were given IP. For 1 × 10^3^ PFU/mouse, the phage and KpnBHU101 were also administered simultaneously.

#### Phage Cocktail in Prophylaxis and Therapy at a Higher Dosage

Two groups, comprising five in each, of the mice were challenged with 100 μL of bacterial suspension containing 8 × 10^7^ CFU/mouse through the intraperitoneal route. After 6 and 24 h of bacterial challenge, 100 μL of phage cocktail containing 1 × 10^12^ PFU/mouse were given IP.

#### Phage Cocktail in Prophylaxis and Therapy at a Constant Dosage

For the bacterial challenge, a dose of 100 μL of KpnBHU101 (8 × 10^7^ CFU/mouse) was used. To analyze the effect of phage in prophylaxis therapy, a constant dose of 100 μL of phage cocktail (1 × 10^5^ PFU/mouse) was given at 5 different time points, namely, simultaneous administered, 6 h before the bacterial challenge, and 6, 12, and 24 h after a bacterial challenge. All the injections were given IP to the respective group having five mice in each. The mice were observed for 96 h. The plan of the phage cocktail administration is shown in Table 1.

#### Determination of Phage Cocktail Dosages Against KpnBHU101 From Blood Circulation

A challenge dose of 100 μL of KpnBHU101 with the concentration 8 × 10^7^ CFU/mouse was given to two groups, comprising five mice each. To analyze the bacterial count in blood circulation after phage therapy, a constant dose of 100 μL of phage cocktail with the concentration 1 × 10^5^ PFU/mouse was given IP at two different time points simultaneously and after 6 h of the bacterial challenge. Then, a repeated dose of phage cocktail was administered once daily till the bacterial count became zero in blood circulation (5 days). The mice were observed for 96 h after the last dose.

The blood was collected in a volume of 100 μL at 30 min, 3, 6, 9, 12, and 24 h after each phage dose. In addition, one mouse was randomly selected for retrobulbar blood collection at a particular time. The blood was used for CFU and for PFU assays. Blood was diluted tenfold, and 100 μL of diluted fluid from each tube was inoculated on MHA plates for spreading and in soft agar tubes for double agar overlay. The plates were incubated at 37°C overnight. The next day, colonies and plaques were counted visually.

#### Estimation of Interleukin-6 and Endotoxin Level in Blood

To estimate the level of cytokines and endotoxin in blood, two groups, comprising five mice each, were challenged with 100 μL of bacterial suspension containing 8 × 10^7^ CFU/mouse through the intraperitoneal route. Six hours after the bacterial challenge, 100 μL of phage cocktail containing 1 × 10^5^ PFU/mouse (group I) and 1 × 10^12^ PFU/mouse (group II) was given IP.

Blood was collected in a volume of 100 μL at 30 min, 3, 6, 9, and 24 h after bacteriophage dose from each group. The serum was separated, and endotoxin and cytokine (pro-inflammatory, IL-6) levels were estimated by ELISA kit (ImmunoTag, G-Biosciences 9800, Page Avenue, St. Louis, MO, United States).

## Results

### Antibiotic Sensitivity Test and Phage Efficiency Against Clinical Strains

The antibiotic sensitivity testing of the recommended antibiotics for clinical use revealed that KpnBHU101 was resistant to all the antibiotics tested, including imipenem, meropenem, ertapenem, polymyxin B, and colistin. Therefore, KpnBHU101 was used for further experiments.

Overall, 50 bacteriophages were isolated from different water sources against KpnBHU101 clinical multidrug-resistant *K. pneumoniae* and designated as ɸKpBHU1 to ɸKpBHU50. Three phages, ɸKpBHU4, ɸKpBHU7, and ɸKpBHU14, were found effective in lysing 77.14%, 71.42 %, and 71.14% of the clinical isolates, respectively. However, these phages were inactive against *Pseudomonas aeruginosa*, *Escherichia coli*, *Salmonella* Typhi, *Acinetobacter lwoffii*, *Enterobacter cloacae*, and *Staphylococcus aureus*.

### Phage Characterization

#### Morphology of Bacteriophages

**FIGURE 1:**
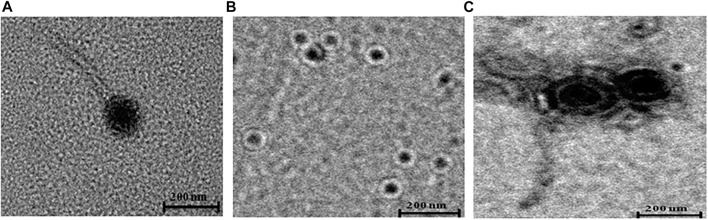
Transmission electron micrographs of phages. ɸKpBHU4 **(A)** and ɸKpBHU14 **(C)** belong to Siphoviridae family, and ɸKpBHU7 **(B)** belongs to Tectiviridae family. The bar indicates 200 nm.

The morphology of bacteriophages ɸKpBHU4, ɸKpBHU7, and ɸKpBHU14 was studied using transmission electron microscopy CRYO-TEM (TALOS S) (Figures 1A–C). Phage ɸKpBHU4 and ɸKpBHU14 had an isometric capsid and long non-contractile tail. Therefore, they may be assigned to the Siphoviridae family based on morphological characteristics. The third phage ɸKpBHU7 had an isometric capsid without a tail assigned to the Tectiviridae family.

#### Characterization of Phages

The lytic property of the phages ɸKpBHU4 and ɸKpBHU14 was preserved up to 48 h at 60°C while phage ɸKpBHU7 activity diminished in the same condition, albeit it was active at 50°C for 48 h. Similarly, these phages were active at a pH range of 4.0–9.0. However, phage ɸKpBHU14 could retain its lytic activity at pH 3.0 also.

**FIGURE 2:**
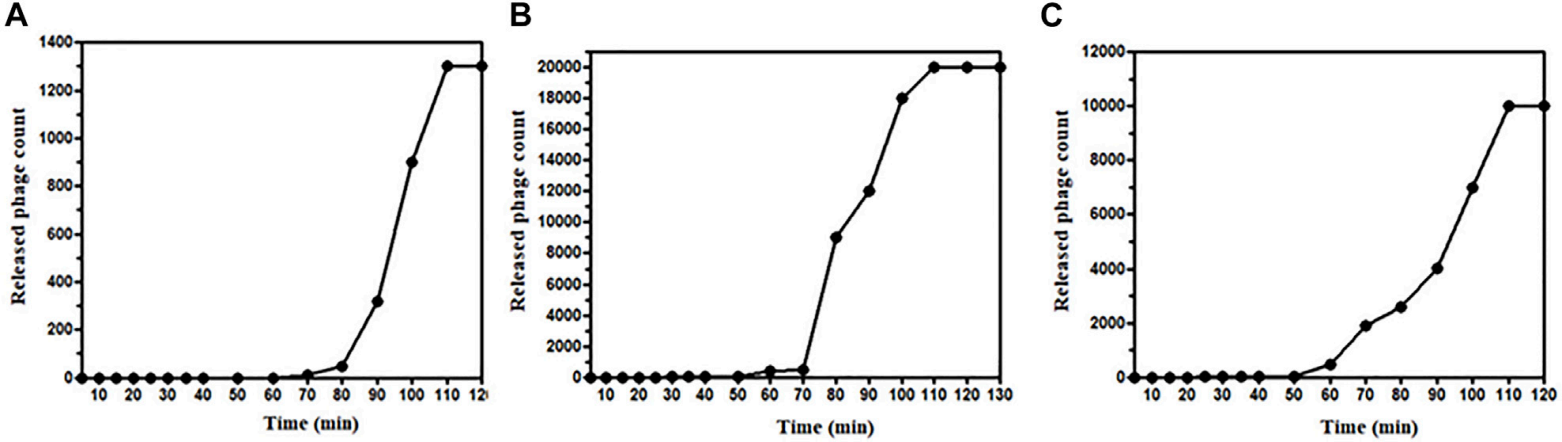
One-step growth curve of bacteriophages; ɸKpBHU4 **(A)**, ɸKpBHU7 **(B)**, ɸKpBHU14 **(C)** for estimation of burst size.

The latent period and burst size of phages ɸKpBHU4, ɸKpBHU7, and ɸKpBHU14 were determined (Figures 2A–C). The latent period for phage ɸKpBHU4 was 30 min; for ɸKpBHU7, it was 70 min and for ɸKpBHU14, it was 25 min. The burst size was approximately 76 PFU/bacterial cells for ɸKpBHU4, 43 PFU/bacterial cells for ɸKpBHU7, and 83 PFU/bacterial cells for ɸKpBHU14 (Figures 2A–C).

**FIGURE 3:**
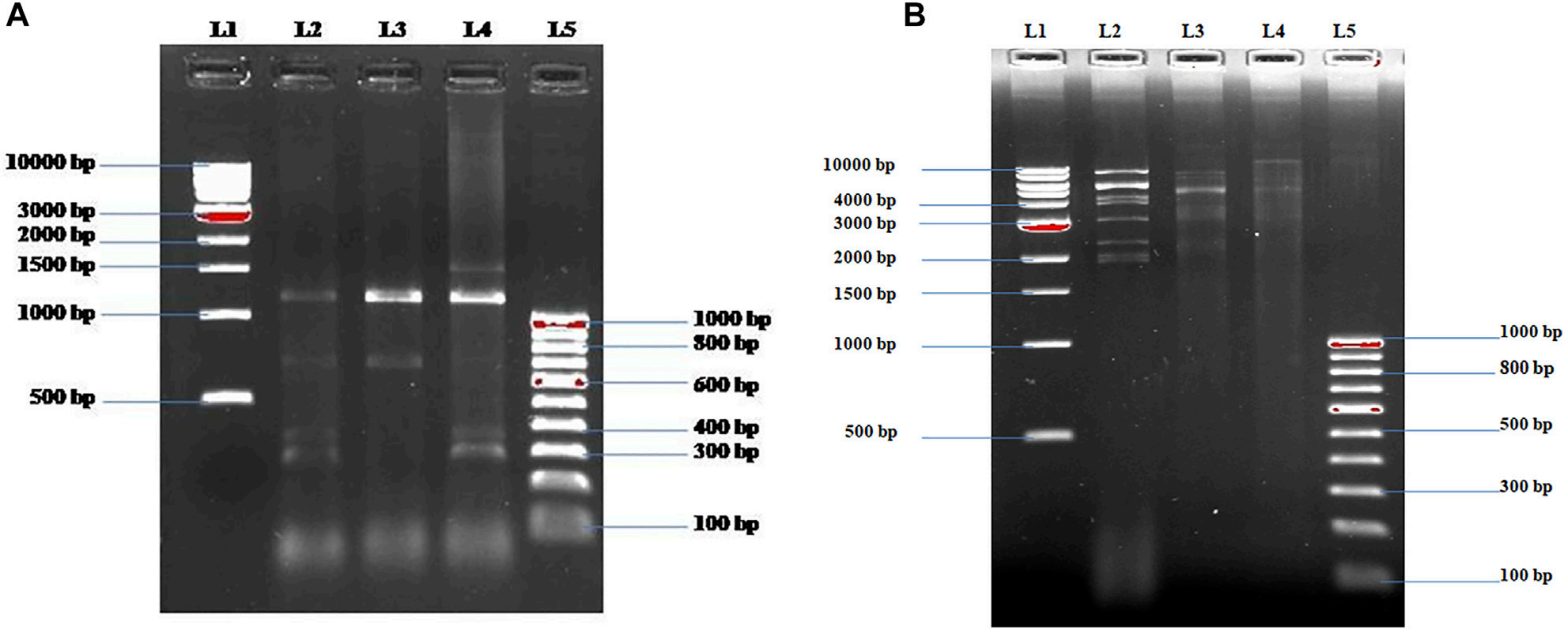
**(A)** Fingerprinting of ɸKpBHU4, ɸKpBHU7, and ɸKpBHU14 bacteriophages genomic DNA with RAPD PCR. Lane-1 (L1): molecular marker (1 kb); lane-2 (L2): ɸKpBHU4; lane-3 (L3): ɸKpBHU7; lane-4 (L4): ɸKpBHU14; and lane-5 (L5): molecular marker (100 bp). **(B)** Restriction endonuclease digestion of bacteriophages genomic DNA. The phage DNA was digested with restriction enzyme EcoRI and run on 1% agarose gel. Lane-1: molecular marker (1 kb) (L1); lane-2 ɸKpBHU4 (L2): lane-3 ɸKpBHU7 (L3): lane-4: ɸKpBHU14 (L4); and lane-5: molecular marker (100 bp) (L5).

The isolated potent phages were further characterized at the genomic level using RAPD-PCR and restriction digestion by isolating their genomic DNA. Different banding patterns were observed in the RAPD-PCR analysis (Figure 3A). Also, the restriction digestion with EcoRI shows a distinct band pattern in all three phages (Figure 3B). The results delineate that the isolated potent phages have different genetic makeup.

#### Animal Studies and Septicemia Model

**FIGURE 4:**
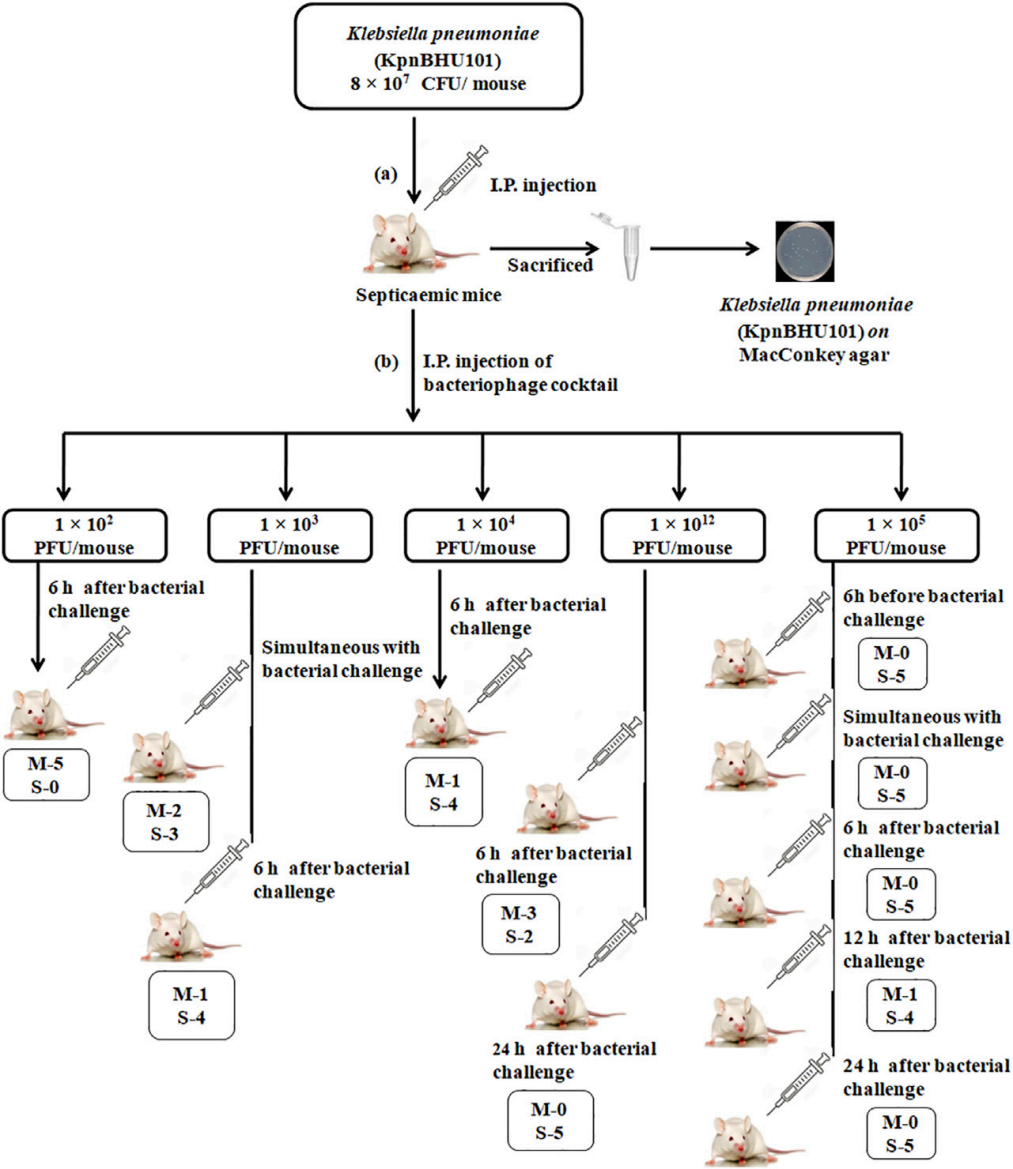
Mouse experiment flowchart **(A)** Depicts the induction of septicemia through intraperitoneal route with *K. pneumoniae* at the dose of 8 × 10^7^ CFU/mouse of KpnBHU101. After 24 h, mice were killed, and organs were homogenized and cultured on MacConkey plates to see the presence of bacteria. **(B)** Outline of the efficacy of the bacteriophage cocktail dosage with varying concentrations at a different time point after the initial bacterial challenge. Each group has 5 mice; (M) depicts the number of dead mice and (S) depicts the number of mice surviving.

The three potent phages showing lytic activity to KpnBHU101 were selected. Furthermore, their cocktail was prepared with an equal concentration of each phage for the *in vivo* evaluation of their efficacy in treating septicemia caused by KpnBHU101 in the mouse model (Figure 4, Supplementary Tables S3.1–S3.5). The LD100 was observed to be 8 × 10^7^ CFU/mouse of KpnBHU101.

#### The Effect of Phages Cocktail in the Developed Septicemia Mice Model

##### Assessment of Phage Efficacy With the Increasing Log of Phage Count

**FIGURE 5:**
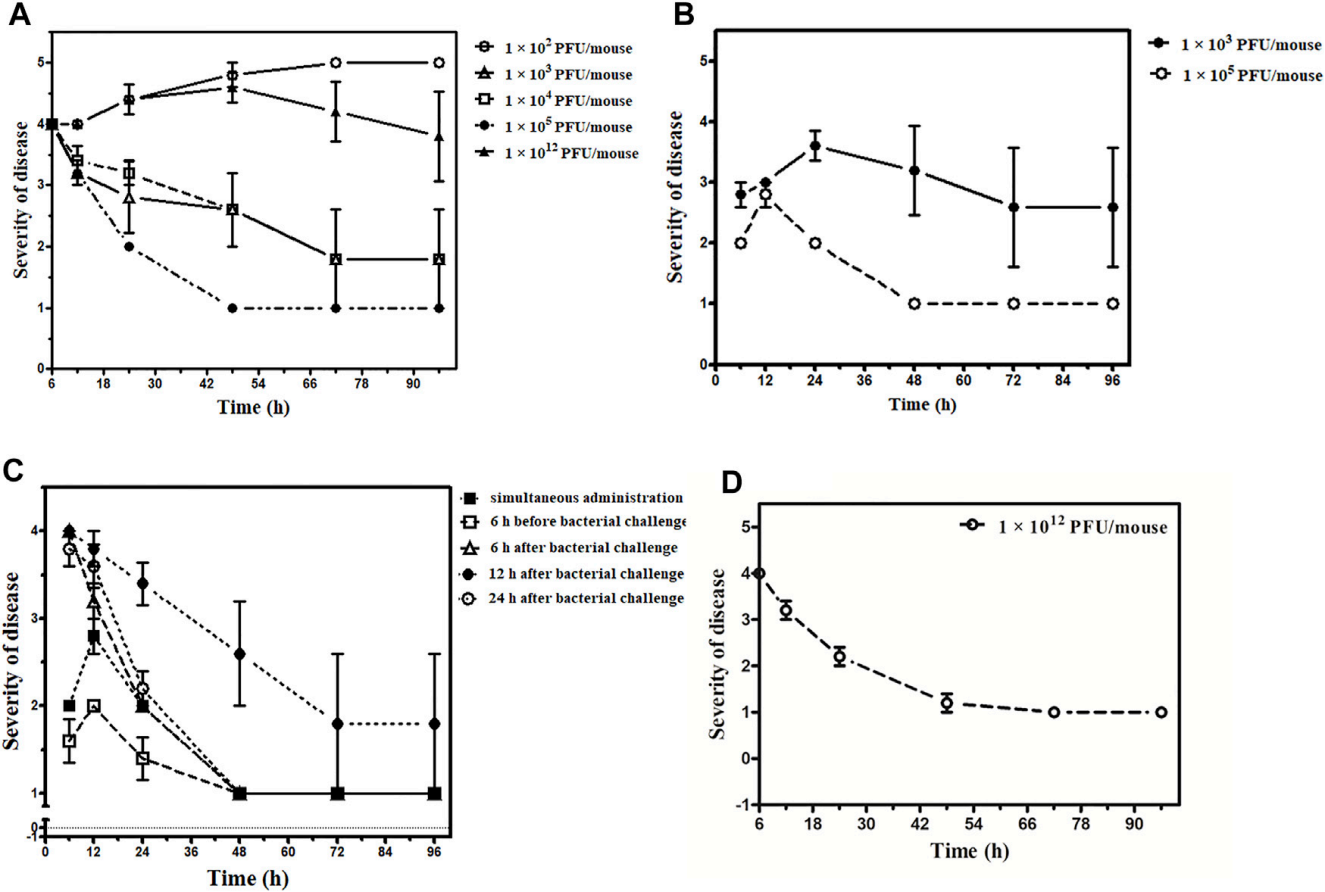
Outcome of the phage therapy on *Klebsiella pneumoniae* septicemia at different time points and phage dosage. The *X*-axis represents the time point of assessment after administration of phage cocktail and *Y*-axis shows the grading of severity of disease ranging from 1- normal; 2- slight illness, lethargy, ruffled fur; 3- moderate illness, severe lethargy, ruffled fur, and hunched back; 4- a severe illness with aforementioned signs, exudative accumulation around eyes; 5- death. **(A)** Phage cocktail (1 × 10^2^, 1 × 10^3^, 1 × 10^4^, 1 × 10^5^, 1 × 10^12^ PFU/mouse) given 6 h after bacterial challenge, 1 × 10^2^ PFU/mouse denoted by an open circle (○), 1 × 10^3^ PFU/mouse denoted by an open triangle (∆), 1 × 10^4^ PFU/mouse denoted by an open square (□), 1 × 10^5^ PFU/mouse denoted by an closed circle (•), 1 × 10^12^ PFU/mouse denoted by an closed triangle (▲). **(B)** Phage cocktail (1 × 10^3^, 1 × 10^5^ PFU/mouse) given simultaneously, 1 × 10^3^ PFU/mouse denoted by the closed circle (•) and 1 × 10^5^ PFU/mouse denoted by an open circle (○). **(C)** Phage cocktail (1 × 10^5^ PFU/mouse) given simultaneously denoted by a closed square (■), 6 h before bacterial challenge denoted by open square (□), 6 h after bacterial challenge denoted by an open triangle (∆), 12 h after bacterial challenge denoted by a closed circle (•), and 24 h after bacterial challenge denoted by an open circle (○). The bacterial challenge dose is constant for the entire group with 8 × 10^7^ CFU/mouse concentration. **(D)** Phage cocktail (1 × 10^12^ PFU/mouse) given 24 h after bacterial challenge denoted by an open circle (○).

Four different concentrations of phage dose, that is, 1 × 10^2^, 1 × 10^3^, 1 × 10^4^, and 1 × 10^5^ PFU/mouse were given to 4 different groups of mice after 6 h of the bacterial challenge. The group received a phage dose of 1 × 10^2^ PFU/mouse of the phage cocktail. All five mice had the signs of severe infection with severe lethargy, ruffled fur, and a hunched back with the exudative accumulated around the eyes at 12 h after the therapy. Four of them were found dead after 48 h. The remaining mice also died after 72 h (Figure 5A, see Supplementary Table S3.1).

The group that received a phage dose of 1 × 10^3^ PFU/mouse had severe illness with severe lethargy, ruffled fur, and a hunched back with the exudative accumulated around the eyes which persisted up to 24 h. Of them, one mouse was found dead at 24 h. However, the rest four became healthy after 72 h. In the group where both phage and bacteria were administered simultaneously, two mice were found dead after 48 h and three mice recovered in 72 h (Figure 5B, see Supplementary Table S3.2).

Furthermore, two of the five mice belonging to the 1 × 10^4^ PFU/mouse phage dosage had increased severity at 12 h. They developed the additional sign of exudative accumulation around the eyes at 24 h. Of them, one mouse was found dead at 48 h. However, the rest of the mice had a severe illness at 24 h but improved afterward; they became normal by 72 h (Figure 5A, see Supplementary Table S3.3).

##### Phage Cocktail in Higher Dosage

The group received a phage dose of 1 × 10^12^ PFU/mouse after 6 h of the bacterial challenge; at 12 h, all five mice were severely ill. All the mice had exudative accumulation around the eyes and severe lethargy, ruffled fur, and hunched back. Two of them died when examined at 24 h. The third mouse was found dead after 72 h. However, the remaining two mice were slightly ill up to 96 h (Figure 5A, see Supplementary Table S3.4), and they became healthy later on. Thus, while the group received a phage dose of 1 × 10^12^ PFU/mouse after 24 h of bacterial challenge, although the illness observed was quite severe at 6 h after therapy, it decreased by 48 h. Furthermore, there was complete recovery when examined at 72 h with no death (Figure 5D, see Supplementary Table S3.4).

##### Phage Cocktail in Constant Dosage

Phage cocktail was given at a constant dose, that is, in the volume of 100 µL containing 1 × 10^5^ PFU/mouse. No mortality occurred in this group or in the simultaneous group. The maximum level of illness went up to grade 3 (moderate disease, severe lethargy, ruffled fur, and hunched back) in four mice after 24 h. However, these symptoms improved by 48 h, and all five became healthy by 72 h (Figures 5B,C, see Supplementary Table S3.5).

In the group that received a phage cocktail 6 h before the bacterial challenge, the maximum level of illness went up to grade 3. All five mice became healthy when observed at 48 h (Figure 5C, see Supplementary Table S3.5). The group received a phage cocktail after 6 h of the bacterial challenge; all five mice were severely lethargic, and had ruffled fur and a hunched back at 24 h. All 5 mice became normal when observed at 72 h (Figure 5C, see Supplementary Table S3.5).

In the group that received phage dose after 12 h of bacterial challenge, the severity of sickness was relatively high, with an average grade point of 4 after 6 h from the start of therapy. The severity of illness increased during 12–48 h from the beginning of the treatment, with one death at 48 h. Later on, at 72 h, the rest of the four mice were seen with improvement, and all of them became healthy by 96 h (Figure 5C, see Supplementary Table S3.5).

In the group that received the phage cocktail after 24 h of bacterial challenge, the severity of illness was high at 6 h after the beginning of therapy. However, it decreased with time, and at 48 h, all the five mice were slightly ill and became normal when observed at 96 h (Figure 5C, see Supplementary Table S3.5).

##### Number of Phage Cocktail Dosage Required for KpnBHU101 Eradication

**FIGURE 6:**
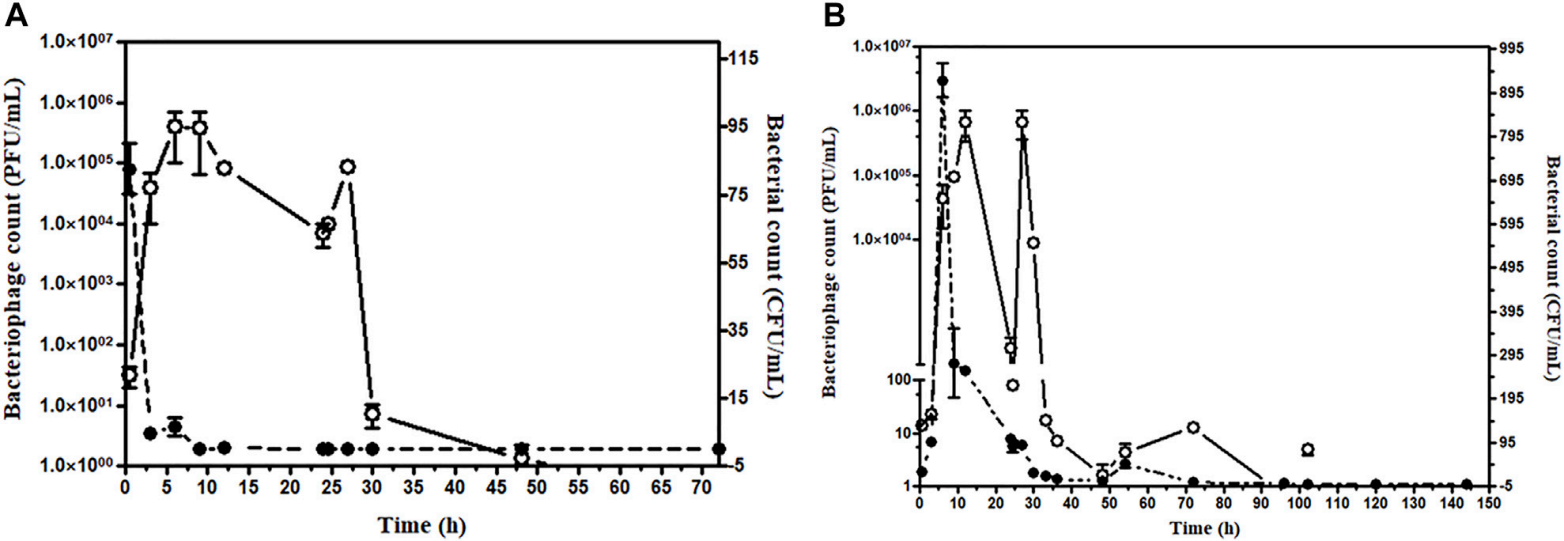
**(A)** Assessment of bacterial and bacteriophage count in the mice blood after simultaneous injection. The bacterial challenge given 8 × 10^7^ CFU/mouse and bacteriophage 1 × 10^5^ PFU/mouse, Open circle (○) denotes PFU/mL, and the closed circle (•) denotes CFU/mL. **(B)** Assessment of bacterial and bacteriophage count in the mice blood up to 144 h bacteriophage cocktail was given 6 h after bacterial challenge. The bacteriophage cocktail was given daily for 5 days. The bacterial challenge given 8 × 10^7^ CFU/mouse and bacteriophage 1 × 10^5^ PFU/mouse. Open circle (○) denotes PFU/mL, and the closed circle (•) denotes CFU/mL.

In the group where bacteria and phage were administered simultaneously, the bacterial count was high at 30 min after simultaneous administration of bacteria and phage. However, the bacterial load decreased and became zero after 24 h. For safety, a second dose of the phage cocktail was given; the bacterial count remained zero after 6 h, and blood remained sterile up to 96 h. However, phage load remains high at 24 h after the first dose of phage, which gradually decreases (Figure 6A).

In the group receiving phage cocktail after 24 h of bacterial challenge, bacterial load was high after 24 h and became low after the first phage dose. However, no bacterium could be isolated from blood after the 6th phage, once-daily dose of the phage cocktail (1 × 10^5^ PFU/mouse). Thus, although mice were recovered after a single phage dose, low bacterial count persisted in blood circulation. The aforementioned result showed that multiple repeated phage dosage is required to eradicate the bacteria from blood circulation (Figure 6B). The efficacy of phage dosage was further corroborated with the complete absence of the bacteria after the dead mouse was analyzed and only phages were present. Furthermore, the peritoneal fluid, liver, spleen, heart, and blood were found to have phages only.

##### Estimation of Cytokine and Endotoxin Levels

**FIGURE 7:**
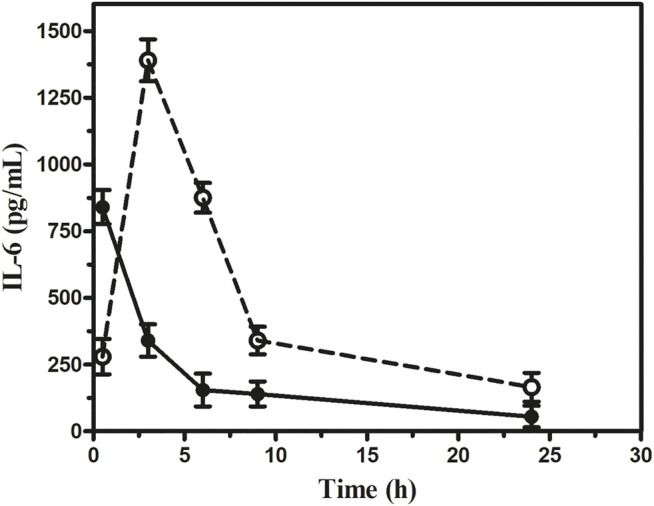
Assessment of interleukin-6 at the different dosages of phage cocktail given 6 h after bacterial challenge. The open circle (**○**) denotes (1 × 10^12^ PFU/mouse), and the closed circle (**•**) denotes (1 × 10^5^ PFU/mouse).

**FIGURE 8:**
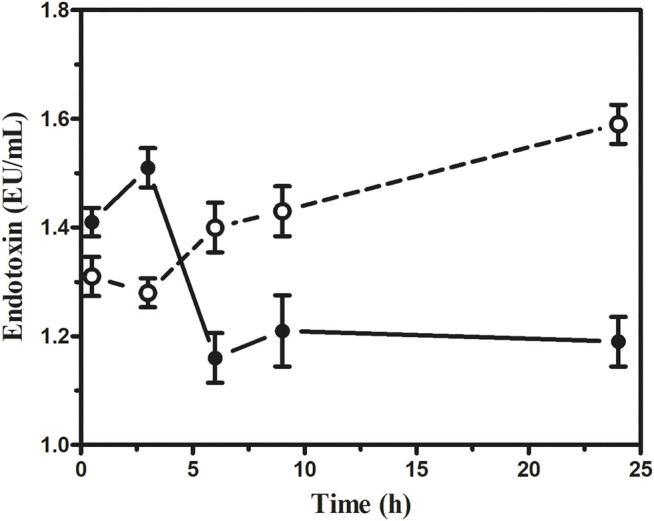
Assessment of endotoxin at the different dosages of phage cocktail given 6 h after bacterial challenge. The open circle (**○**) denotes (1 × 10^12^ PFU/mouse), and the closed circle (•) denotes (1 × 10^5^ PFU/mouse).

Our results indicated that the serum IL-6 level in sepsis mice rose markedly. However, in the 1 × 10^12^ PFU/mouse group, the highest IL-6 level observed was 1,390 pg/ml at 3 h, which gradually decreased to 165 pg/ml at 24 h. On the other hand, the highest IL-6 level observed in the 1 × 10^5^ PFU/mouse group was 840 pg/ml at 30 min, which gradually decreased to 55 pg/ml (Figure 7). In the 1 × 10^12^ PFU/mouse group, the endotoxin level increased after 3 h and overtook 1 × 10^5^ PFU/mouse endotoxin levels at 6 h and gradually increased with the highest endotoxin level of 1.59 endotoxin unit (EU) at 24 h after the administration of the phage cocktail. While the group was given 1 × 10^5^ PFU/mouse, the endotoxin level was 1.45 EU at 3 h, which gradually decreased to the lowest level of 1.19 EU after 24 h. However, the endotoxin level in the mice in 1 × 10^12^ PFU/mouse group remains consistently high up to 24 h compared to those receiving 1 × 10^5^ PFU/mouse (Figure 8, See Supplementary Table S4).

The final outcome of the effect of phage therapy on *K. pneumoniae* septicemia mice model at different time points and dosage was demonstrated in Figure 6, where the gradation of the severity of septicemia-induced mice at specific time points was plotted against the administration of different concentrations of phage cocktail. The result suggests that at different time points ranging from 6 to 24 h, the phage cocktail of concentration 1 × 10^5^ PFU/mouse against the constant bacterial challenge of concentration 8 × 10^7^ CFU/mouse is the safest dose for therapeutic intervention.

## Discussion

The potentials of phage therapy against the emerging multidrug-resistant bacteria, either in planktonic or biofilm forms, seem to be tremendous. Therefore, we made an effort to optimize the concentration of specific phage cocktails per dose and the frequency of dosage required for septicemia of varying severity caused by *K. pneumoniae* in immunocompetent Swiss albino mice in the present study. We observed that 8 × 10^7^ CFU/mouse of *K. pneumoniae* to be the LD100 for Swiss albino mice when injected IP, the deaths took place between 24 and 48 h after the bacterial challenge. Previous studies also have documented a similar LD 100 dose 4 × 107 CFU/mouse of *K. pneumoniae* within 62 h post-bacterial challenge, in mice strain C57BL/6J through intraperitoneal route (Rodrigues et al., 2020).

The mortality of 100, 40, 20, and 0% with the dosage of phage cocktail having phage counts of 1 × 10^2^, 1 × 10^3^, 1 × 10^4^, and 1 × 10^5^ PFU/mouse per mouse, respectively, given 6 h after the bacterial challenge, indicates that only 1 × 10^5^ PFU/mouse was capable of controlling the infection.

Overall, the intraperitoneal administration of 1 × 10^5^ PFU/mouse phage cocktail dose seems safe as the simultaneous, 6, 12, and 24 h post-infection administration resulted in 100% recovery from acute *K. pneumoniae* septicemia. It appears that there were enough phages to kill the bacteria effectively and with the release of a tolerable concentration of bacterial endotoxin. Interestingly, in the case of the dose of 1 × 10^12^ PFU/mouse, when given in the later stages of septicemia, faster recovery could be achieved than the lower dose of 1 × 10^5^ PFU/mouse without any observable adverse effect. On the other hand, 1 × 10^12^ PFU/mouse was associated with mortality when administered at the early onset of the bloodstream infection (6 h after bacterial challenge). Earlier workers had also reported a similar outcome, for example, Hung et al. (2011) observed that when intramuscular injection of 2 × 10^9^ PFU/mouse was given 30 min after *K. pneumoniae* challenge, it caused 100% mortality (Hung et al., 2011). Wang et al. (2006) reported a mortality rate of 60% in BALB/c mice in cases of *E. coli* septicemia when phage administration was delayed by 60 min (Wang et al., 2006). We have a similar experience with *P. aeruginosa* septicemia as a dose of 1 × 10^12^ PFU/mouse given 6 h post-infection resulted in the 60% mortality (Nath et al., 2019). The cause of death at the early stage of infection with higher dosages of phage may be possible because of the zone-like phenomenon, that is, optimum quantity of bacteria and phage in circulation for sudden bacterial lysis. The endotoxin level remained almost twice higher in the group given 1 × 10^12^ PFU/mouse than in mice with 1 × 10^5^ PFU/mouse throughout 24 h after phage injection and deaths at 24 h. The high endotoxin released might be responsible for the fatal outcome. The pro-inflammatory cytokine IL-6 released maximum at 3 h with a 1 × 10^12^ PFU/mouse dose and came down very fast. Therefore, cytokine storms may not be the reason for the deaths in this group. However, IL-6 remained constant in the mice group on 1 × 10^5^ PFU/mouse than in 1 × 10^12^ PFU/mouse. On the contrary, the endotoxin level in the group receiving 1 × 10^12^ PFU/mouse, although it increased later, remained consistently higher than that on 1 × 10^5^ PFU/mouse. It implies therefore that a higher level of endotoxin in the group on 1 × 10^12^ PFU/mouse might be the cause of death due to the massive host bacterial lysis by phages.

We have carried out the CFU counts of bacteria and PFU counts of phage at different time points of therapy, picking up one mouse randomly from each of the different groups. However, a single dose of phage cocktail of 1 × 10^5^ PFU/mouse prevented the deaths. However, few colonies of *K. pneumoniae* could be grown 24 h post-phage administration*.* Probably the remaining lower counts of circulating bacteria are effectively dealt with by the innate immune system of the immunocompetent hosts. However, six repeated dosages of phage cocktails at the quantity of 1 × 10^5^ PFU/mouse resulted in the sterilization of the peripheral blood. Seemingly, a single phage dose may be inadequate to eradicate the infecting bacteria in an immunocompromised host. This speculation implies that repeated dosage of phage cocktails must be ensured in immunocompromised hosts to eradicate the invading bacteria.

If we extrapolate the 1 × 10^5^ PFU/mouse dose weighing 25 gm with a human weighing 60 kg, the safe dose will be approximately 1 × 10^8^ PFU/person. However, in children, the dose may be reduced according to body weight. The other worth mentioning observation is that no resistant mutants developed during therapy with the cocktail of three phages. This finding indicates that the cocktail took care of mutants if they arose during the short duration of the present treatment regime. However, the genome sequencing of both bacteria and individual phage of the cocktail could further add strength to the manuscript.

## Conclusion

The present study provides preliminary data regarding interleukins, endotoxins, circulating bacteria, and bacterial viruses. However, it would be optimum to include more mice in each group to have robust biochemical data during therapy. Furthermore, all five mice should be bled to estimate the aforementioned biochemical parameters to avoid individual variations at a particular point in time.

The optimized dosage of phage cocktails and the maintenance of vitals (blood pressure, oxygen saturation, etc.) in clinical cases may provide encouraging results in cases of difficult septicemia with different severity of infection. However, it will be worth emphasizing that dosage in the multiplicity of infection (MOI) concept will not work in clinical cases. In addition, it is challenging to quantify the bacteria in circulation at a particular time in the patients (Abedon, 2016). Thus, phage therapy will be a boon in life-threatening septicemia caused by resistant bacteria, including ESKAPE organisms in the era of antimicrobial resistance, if given at an optimized dosage of the cocktail.

## Data Availability

The original contributions presented in the study are included in the article/Supplementary Material; further inquiries can be directed to the corresponding author.

## References

[B1] Abedon S. T. (2016). Phage Therapy Dosing: The Problem(s) with Multiplicity of Infection (MOI). Bacteriophage 6 (3), e1220348. 10.1080/21597081.2016.1220348 27738558 PMC5056779

[B2] Adams M. H. (1959). Bacteriophages. New York: Interscience Publishers.

[B3] Arnold R. S. Thom K. A. Sharma S. Phillips M. Kristie Johnson J. Morgan D. J. (2011). Emergence of *Klebsiella pneumoniae* Carbapenemase-Producing Bacteria. South. Med. J. 104 (1), 40–45. 10.1097/SMJ.0b013e3181fd7d5a 21119555 PMC3075864

[B4] Capone A. Giannella M. Fortini D. Giordano A. Meledandri M. Ballardini M. (2013). High Rate of Colistin Resistance Among Patients with Carbapenem-Resistant *Klebsiella pneumoniae* Infection Accounts for an Excess of Mortality. Clin. Microbiol. Infect. 19 (1), E23–E30. 10.1111/1469-0691.12070 23137235

[B5] Czajkowski R. Ozymko Z. de Jager V. Siwinska J. Smolarska A. Ossowicki A. (2015). Genomic, Proteomic and Morphological Characterization of Two Novel Broad Host Lytic Bacteriophages ΦPD10.3 and ΦPD23.1 Infecting Pectinolytic Pectobacterium Spp. And Dickeya Spp. PLOS ONE 10 (3), e0119812. 10.1371/journal.pone.0119812 25803051 PMC4372400

[B6] Gangwar M. Rastogi S. Singh D. Shukla A. Dhameja N. Kumar D. (2021). Study on the Effect of Oral Administration of Bacteriophages in Charles Foster Rats with Special Reference to Immunological and Adverse Effects. Front. Pharmacol. 12, 615445. 10.3389/fphar.2021.615445 33912038 PMC8072658

[B7] Humphries R. M. Ambler J. Mitchell S. L. Castanheira M. Dingle T. Hindler J. A. (2018). CLSI Methods Development and Standardization Working Group Best Practices for Evaluation of Antimicrobial Susceptibility Tests. J. Clin. Microbiol. 56 (4), e01934–01917. 10.1128/JCM.01934-17 29367292 PMC5869819

[B8] Hung C. H. Kuo C. F. Wang C. H. Wu C. M. Tsao N. (2011). Experimental Phage Therapy in Treating Klebsiella Pneumoniae-Mediated Liver Abscesses and Bacteremia in Mice. Antimicrob. Agents Chemother. 55 (4), 1358–1365. 10.1128/AAC.01123-10 21245450 PMC3067190

[B9] Kesik-Szeloch A. Drulis-Kawa Z. Weber-Dabrowska B. Kassner J. Majkowska-Skrobek G. Augustyniak D. (2013). Characterising the Biology of Novel Lytic Bacteriophages Infecting Multidrug Resistant *Klebsiella pneumoniae* . Virol. J. 10, 100. 23537199 10.1186/1743-422X-10-100 PMC3620542

[B10] Manohar P. Tamhankar A. J. Lundborg C. S. Nachimuthu R. (2019). Therapeutic Characterization and Efficacy of Bacteriophage Cocktails Infecting *Escherichia coli*, *Klebsiella pneumoniae*, and Enterobacter Species. Front. Microbiol. 10 (574), 574. 10.3389/fmicb.2019.00574 30949158 PMC6437105

[B11] Nath G. Janam R. Kumar R. Gangwar M. (2019). Bacteriophage Therapy: An Alternative to Antibiotics-An Experimental Study in MiceAn Alternative to Antibiotics—An Experimental Study in Mice. Ann. Natl. Acad. Med. Sci. (India) 55 (03), 151–158. 10.1055/s-0039-1698545

[B12] Patel J. B. Cockerill F. Bradford P. A. (2015). Performance Standards for Antimicrobial Susceptibility Testing. twenty-fifth informational supplement.

[B13] Prins J. M. van Deventer S. J. Kuijper E. J. Speelman P. (1994). Clinical Relevance of Antibiotic-Induced Endotoxin Release. Antimicrob. Agents Chemother. 38 (6), 1211–1218. 10.1128/aac.38.6.1211 8092816 PMC188188

[B14] Rodrigues M. X. Yang Y. de Souza Meira E. B. Jr do Carmo Silva J. Bicalho R. C. (2020). Development and Evaluation of a New Recombinant Protein Vaccine (YidR) against *Klebsiella pneumoniae* Infection. Vaccine 38 (29), 4640–4648. 10.1016/j.vaccine.2020.03.057 32444194

[B15] Rudd K. E. Johnson S. C. Agesa K. M. Shackelford K. A. Tsoi D. Kievlan D. R. (2020). Global, Regional, and National Sepsis Incidence and Mortality, 1990-2017: Analysis for the Global Burden of Disease Study. Lancet 395 (10219), 200–211. 10.1016/S0140-6736(19)32989-7 31954465 PMC6970225

[B16] Sadekuzzaman M. Yang S. Mizan M. F. R. Ha S. D. (2017). Reduction of *Escherichia coli* O157:H7 in Biofilms Using Bacteriophage BPECO 19. J. Food Sci. 82 (6), 1433–1442. 10.1111/1750-3841.13729 28542913

[B17] Schooley R. T. Biswas B. Gill J. J. Hernandez-Morales A. Lancaster J. Lessor L. (2017). Development and Use of Personalized Bacteriophage-Based Therapeutic Cocktails to Treat a Patient with a Disseminated Resistant Acinetobacter Baumannii Infection. Antimicrob. Agents Chemother. 61 (10). 10.1128/AAC.00954-17 PMC5610518 28807909

[B18] Skorup P. Maudsdotter L. Lipcsey M. Larsson A. Sjölin J. (2020). Mode of Bacterial Killing Affects the Inflammatory Response and Associated Organ Dysfunctions in a Porcine *E. coli* Intensive Care Sepsis Model. Crit. Care 24 (1), 646. 10.1186/s13054-020-03303-9 33189146 PMC7666448

[B19] Taha O. A. Connerton P. L. Connerton I. F. El-Shibiny A. (2018). Bacteriophage ZCKP1: A Potential Treatment for *Klebsiella pneumoniae* Isolated from Diabetic Foot Patients. Front. Microbiol. 9 (2127), 2127. 10.3389/fmicb.2018.02127 30254618 PMC6141743

[B20] Wang J. Hu B. Xu M. Yan Q. Liu S. Zhu X. (2006). Therapeutic Effectiveness of Bacteriophages in the rescue of Mice with Extended Spectrum Beta-Lactamase-Producing *Escherichia coli* Bacteremia. Int. J. Mol. Med. 17 (2), 347–355. 10.3892/ijmm.17.2.347 16391836

[B21] Zurabov F. Zhilenkov E. (2021). Characterization of Four Virulent *Klebsiella pneumoniae* Bacteriophages, and Evaluation of Their Potential Use in Complex Phage Preparation. Virol. J. 18 (1), 9. 10.1186/s12985-020-01485-w 33407669 PMC7789013

