# Supplementary material for: Evaluation of Bacteriophage Cocktail on Septicemia Caused by Colistin-Resistant Klebsiella pneumoniae in Mice Model
Source: Front Pharmacol. 2022 Feb 7;13:778676. doi: 10.3389/fphar.2022.778676 (PMC8860340; doi:10.3389/fphar.2022.778676)
Supplement: Supplementary file 1 [file DataSheet1.PDF]

Supplementary File

Table S1. Antibiotics used for antimicrobial susceptibility testing of *Klebsiella pneumoniae* (KpnBHU101)

**S.N.**	**Antibiotic disc**	**Susceptibility range (mm)**	**Sensitivity pattern**
1	Ampicillin (10mcg)	≥17	0/R
2	Amoxicillin (20mcg) + Clavulanic acid(10mcg)	≥18	0/R
3	Cefazolin/Cephalexin(30 mcg)	≥18	0/R
4	Cefuroxime (30 mcg),	≥18	0/R
5	Cefotaxime/Ceftriaxone (30 mcg)	≥26	0/R
6	Cefpirome/Cefepime (30 mcg),	≥18	0/R
7	Gentamicin (10 mcg)	≥15	0/R
8	Amikacin (30 mcg)	≥17	0/R
9	Ciprofloxacin (5 mcg)	≥21	0/R
10	Levofloxacin (5 mcg)	≥19	0/R
11	Trimethoprim (1.25mcg) + Sulfamethoxazole (23.75 mcg)	≥16	0/R
12	Meropenem (10 mcg)	≥16	0/R
13	Imipenem (10 mcg)	≥16	0/R
14	Ertapenem (10 mcg)	≥22	0/R
16.	Colistin (µg/µL)	≥ 2µg/µL	3.22µg/µL/R

Table-S2. Determination of LD100 in *Klebsiella pneumoniae* septicemia mice model

**Experiment group**	**Number of mouse**	**Bacterial count (CFU/mouse)/100 uL**	**Survival rate (%)**	**Mortality rate (%)**
Control group	5	Normal saline	100%	0%
I	5	3 x 10^7	100%	0%
II	5	5 x 10^7	100%	0%
III	5	7 x 10^7	60%	40%
IV	5	8 x 10^7	0	100% (within 24-48 h)
V	5	1 x 10^8	0	100% (within 16-17 h)

Table S3.1. The effect of phage cocktail with 1 x 10^2 PFU/mouse in different septicaemia mice experimental groups.

Mice group	Phage cocktail with 1 x 10^2 PFU/mouse	Observed severity in septicaemia model after intervention with phage cocktail at different time point (h)					
		6	12	24	48	72	96
I	Phage cocktail 6 h after bacterial challenge	4+4+4+4+4 (4.0)	4+4+4+4+4 (4.0)	4+4+4+5+5 (4.4)	4+5+5+5+5 (4.8)	5+5+5+5+5 (5.0)	5+5+5+5+5 (5.0)

Table S3.2. The effect of phage cocktail with 1x10^3 PFU/mouse in different septicaemia mice experimental groups

Mice group	Phage cocktail with 1 x 10^3 PFU/mouse	Observed severity in septicaemia model after intervention with phage cocktail at different time point (h)					
		6	12	24	48	72	96
I	Simultaneous administration of phage and KpnBHU101	3+3+3+3+2 (2.8)	3+3+3+3+3 (3.0)	3+4+4+4+3 (3.6)	2+5+5+2+2 (3.2)	5+5+1+1+1 (2.6)	5+5+1+1+1 (2.6)
II	Phage cocktail 6 h after bacterial challenge	4+4+4+4+4 (4.0)	3+3+3+3+4 (3.2)	3+2+2+2+5 (2.8)	2+2+2+2+5 (2.6)	1+1+1+1+5 (1.8)	1+1+1+1+5 (1.8)

Table S3.3. The effect of phage cocktail with 1x10^4 PFU/mouse in different septicaemia mice experimental groups

Mice group	Phage cocktail with 1 x 10^4 PFU/mouse	Observed severity in septicaemia model after intervention with phage cocktail at different time point (h)					
		6	12	24	48	72	96
I	Phage cocktail 6 h after bacterial challenge	4+4+4+4+4 (4.0)	3+3+3+4+4 (3.4)	3+3+3+3+4 (3.2)	2+2+2+2+5 (2.6)	1+1+1+1+5 (1.8)	1+1+1+1+5 (1.8)

Table S3.4. The effect of phage cocktail with 1x10^{12} PFU/mouse in different septicaemia mice experimental groups

Mice group	Phage cocktail with 1 x 10^{12} PFU/mouse	Observed severity in septicaemia model after intervention with phage cocktail at different time point (h)					
		6	12	24	48	72	96
I	Phage cocktail 6 h after bacterial challenge	4+4+4+4+4 (4.0)	4+4+4+4+4 (4.0)	4+4+4+5+5 (4.4)	5+4+4+5+5 (4.6)	5+3+3+5+5 (4.2)	5+2+2+5+5 (3.8)
II	Phage cocktail 24 h after bacterial challenge	4+4+4+4+4 (4.0)	3+4+3+3+3 (3.2)	2+3+2+2+2 (2.2)	1+2+1+1+1 (1.2)	1+1+1+1+1 (1.0)	1+1+1+1+1 (1.0)

Table S3.5. The effect of phage cocktail with constant dose of $1x10^5$ PFU/mouse in different septicaemia mice experimental groups.

Mice group	Phage cocktail with 1 x 10^5 PFU/mouse	Observed severity in septicaemia model after intervention with Phage cocktail at different time point (h)					
		6	12	24	48	72	96
I	Simultaneous administration of phage and KpnBHU101	2+2+2+2+2 (2.0)	2+3+3+3+3 (2.8)	2+2+2+2+2 (2.0)	1+1+1+1+1 (1.0)	1+1+1+1+1 (1.0)	1+1+1+1+1 (1.0)
II	Phage cocktail 6 h before bacterial challenge	1+2+2+1+2 (1.6)	2+2+2+2+2 (2.0)	1+1+2+1+2 (1.4)	1+1+1+1+1 (1.0)	1+1+1+1+1 (1.0)	1+1+1+1+1 (1.0)
III	Phage cocktail 6 h after bacterial challenge	4+4+4+4+4 (4.0)	3+4+3+3+3 (3.2)	2+2+2+2+2 (2.0)	1+1+1+1+1 (1.0)	1+1+1+1+1 (1.0)	1+1+1+1+1 (1.0)
IV	Phage cocktail 12 h after bacterial challenge	4+4+4+4+4 (4.0)	4+3+4+4+4 (3.8)	3+3+3+4+4 (3.4)	2+2+2+5+2 (2.6)	1+1+1+5+1 (1.8)	1+1+1+5+1 (1.8)
V	Phage cocktail 24 h after bacterial challenge	4+4+4+4+3 (3.8)	4+4+4+3+3 (3.6)	3+2+2+2+2 (2.2)	1+1+1+1+1 (1.0)	1+1+1+1+1 (1.0)	1+1+1+1+1 (1.0)

1 – Normal; 2 – Slight illness, lethargy, ruffled fur; 3 – Moderate illness, severe lethargy, ruffled fur and hunched back; 4- Severe illness with above sign, exudative accumulation around eyes; 5- Death; Figure in parenthesis shows the average of the signs of all the 5 mice in a particular study group.

Table S4. Endotoxin and Il-6 level in mice blood after administration of phage cocktail.

**Time**	**Mice (control)**		***K. pneumoniae* (control)**		**Phage 10^5 PFU/mouse (control)**		**Phage 10^{12} PFU/mouse (control)**		**Phage 10^5 PFU/mouse**		**Phage 10^{12} PFU/mouse**	
	**Endotoxin (EU)**	**IL-6 pg/mL**	**Endotoxin (EU)**	**IL-6 pg/mL**	**Endotoxin (EU)**	**IL-6 pg/mL**	**Endotoxin (EU)**	**IL-6 pg/mL**	**Endotoxin (EU)**	**IL-6 pg/mL**	**Endotoxin (EU)**	**IL-6 pg/mL**
**0.5 h**	-	-	-	-	-	-	-	-	1.41	840	1.31	280
**3 h**	-	-	-	-	-	-	-	-	1.51	340	1.28	1390
**6 h**	0	0	0.1	135	0	0	0.1	0	1.16	150	1.4	875
**9 h**	-	-	-	-	-	-	-	-	1.21	140	1.43	340
**24 h**	0	0	0.71	210	0	0	0	0	1.16	55	1.59	165

The symbol **(-)** represents the blood was not withdrawn at that time point.
